# Application of Machine Learning for the Prediction of Etiological Types of Classic Fever of Unknown Origin

**DOI:** 10.3389/fpubh.2021.800549

**Published:** 2021-12-24

**Authors:** Yongjie Yan, Chongyuan Chen, Yunyu Liu, Zuyue Zhang, Lin Xu, Kexue Pu

**Affiliations:** ^1^School of Medical Informatics, Chongqing Medical University, Chongqing, China; ^2^Key Laboratory of Data Engineering and Visual Computing, Chongqing University of Posts and Telecommunications, Chongqing, China; ^3^Medical Records and Statistics Office, The Second Affiliated Hospital of Chongqing Medical University, Chongqing, China

**Keywords:** fever of unknown origin, machine learning, etiology, retrospective analysis, LightGBM algorithm

## Abstract

**Background:** The etiology of fever of unknown origin (FUO) is complex and remains a major challenge for clinicians. This study aims to investigate the distribution of the etiology of classic FUO and the differences in clinical indicators in patients with different etiologies of classic FUO and to establish a machine learning (ML) model based on clinical data.

**Methods:** The clinical data and final diagnosis results of 527 patients with classic FUO admitted to 7 medical institutions in Chongqing from January 2012 to August 2021 and who met the classic FUO diagnostic criteria were collected. Three hundred seventy-three patients with final diagnosis were divided into 4 groups according to 4 different etiological types of classical FUO, and statistical analysis was carried out to screen out the indicators with statistical differences under different etiological types. On the basis of these indicators, five kinds of ML models, i.e., random forest (RF), support vector machine (SVM), Light Gradient Boosting Machine (LightGBM), artificial neural network (ANN), and naive Bayes (NB) models, were used to evaluate all datasets using 5-fold cross-validation, and the performance of the models were evaluated using micro-F1 scores.

**Results:** The 373 patients were divided into the infectious disease group (*n* = 277), non-infectious inflammatory disease group (*n* = 51), neoplastic disease group (*n* = 31), and other diseases group (*n* = 14) according to 4 different etiological types. Another 154 patients were classified as undetermined group because the cause of fever was still unclear at discharge. There were significant differences in gender, age, and 18 other indicators among the four groups of patients with classic FUO with different etiological types (*P* < 0.05). The micro-F1 score for LightGBM was 75.8%, which was higher than that for the other four ML models, and the LightGBM prediction model had the best performance.

**Conclusions:** Infectious diseases are still the main etiological type of classic FUO. Based on 18 statistically significant clinical indicators such as gender and age, we constructed and evaluated five ML models. LightGBM model has a good effect on predicting the etiological type of classic FUO, which will play a good auxiliary decision-making function.

## Introduction

Fever of unknown origin (FUO) is a difficult and active medical topic in the diagnosis and treatment of difficult and complicated diseases in internal medicine, and it is a challenging problem for physicians (1, 2). Currently, there are four categories of FUOs: classic FUO, FUO in hospitalized patients, FUO in patients with agranulocytosis, and FUO in patients with human immunodeficiency virus (HIV) infection (3, 4). Among them, classic FUO is the most common, which is defined as a disease that lasts for >3 weeks, has a body temperature of >38.3°C at least three times, and cannot be diagnosed after systematic and comprehensive examinations in the outpatient or inpatient department of the hospital for >1 week (5, 6). There are >200 kinds of causes of classic FUO (7). For clinicians, because of its complex etiology, lack of characteristic clinical signs, and inadequate laboratory tests, the diagnosis is very difficult (8). The etiological categories of classic FUO are infectious disease, non-infectious inflammatory disease (NIID), neoplastic disease, and others, and the treatment methods vary greatly, including anti-infective drugs, hormones, and chemotherapy (9–11). With the development of immunohistopathology and modern imaging (12, 13), the diagnosis of classic FUO has become easier, but the final diagnosis is often difficult and up to 50% of cases cannot be confirmed (8, 11, 14, 15).

The diagnostic process of a classic FUO includes four steps: to determine whether it belongs to classic FUO, a first stage of primary screening, a second stage of specific examination, and treatment (including symptomatic and diagnostic treatment) (4). Among them, the first stage (etiological screening) includes improving medical history collection, physical examination, and non-invasive laboratory and auxiliary examinations in line with local medical standards. After the first stage of screening, some patients are diagnosed and some patients offer no diagnostic clues and enter the second stage, which requires further specific examinations. The second phase of the process is more complex, partly invasive, and more expensive. Therefore, the first stage of etiology screening is very important. If the etiology of a FUO can be classified into one category, no matter the disease that caused the FUO, the direction of diagnosis can be determined, which is of great significance to physicians (16, 17). Previous studies of classic FUO have focused on the etiology, prognosis, or diagnosis of classic FUO (18, 19). So far, few researchers have studied the etiological causes of classic FUO from the perspective of clinical prediction models and machine learning (ML) (16). In recent years, ML has been widely used in the medical field and has achieved good results in disease diagnosis, risk assessment, and other factors (20–22).

In this study, the clinical data and etiological types of classic FUO patients were retrospectively analyzed, and a predictive model of FUO etiology was established to help clinicians make reasonable decisions in the diagnosis of classic FUO, improve diagnostic accuracy, and reduce the misdiagnosis rate.

## Materials and Methods

### Materials

The clinical data of 527 patients with classic FUO admitted to seven medical institutions in Chongqing from January 2012 to August 2021 were selected. The selected patients, whose ages ranged from 14 years old upwards, had each been hospitalized for more than a week with a fever higher than 38.3°C (101°F) that had occurred on several occasions and had persisted for at least 21 days (4, 8). Patients diagnosed with HIV infection before hospitalization, patients with immunodeficiency disorders, and pregnant women were screened out (4, 8). Of the 527 patients with classic FUO, 373 were finally diagnosed and 154 were not diagnosed at discharge. A total of 373 patients with classic FUO were divided into four groups according to their diagnosis and medical record information: infectious disease, NIID, neoplastic disease, and other diseases groups.

The index system of this study included general information (gender and age), past history (operation history and history of blood transfusion), accompanying symptoms (headache/consciousness disorders, nasal obstruction, sore throat, abdominal pain, arthralgia, muscle pain, and rash), physical (lymphadenopathy, hepatomegaly, and splenomegaly) and laboratory examinations [globulin, red blood cell (RBC), lactate dehydrogenase (LDH), C-reactive protein (CRP), procalcitonin (PCT), erythrocyte sedimentation rate (ESR), monocyte, basophils, eosinophils, lymphocyte, white blood cell (WBC), alkaline phosphatase (ALP), platelet (PLT), alanine aminotransferase (ALT), aspartate aminotransferase (AST), and gamma-glutamyltransferase (GGT)], and the final diagnosis of etiological types.

The research protocol was approved by the Medical Research Ethics Committee of Chongqing Medical University.

### Statistical Analysis

SPSS 25.0 statistical software was used for data processing. The continuity index was analyzed with a normality test, the median (M) and quartile (P_25_, P_75_) were used to express a non-normal distribution, the Kruskal–Wallis test was used to compare between groups. The normal distribution was expressed by *x* ± s. The analysis of variance was used to compare multiple groups, and the least significance difference (LSD) method was used for comparisons between two groups. The classification index was expressed by rate (%), and the comparison between groups was performed with a χ^2^-test. *P* < 0.05 was considered statistically significant. We used Python (version 3.7.3) for algorithm development.

### Machine Learning

This study was based on the aforementioned differences that were statistically significant indicators to build the model. In order to determine the best model for classifying etiological types in this study, we compared the performance of the following representative ML classification algorithms: RF, SVM, LightGBM, ANN, and NB. For each algorithm, we used the 5-fold cross-validation method to split the data, each time using the training set to train the model and verify the performance of the model on the test set data. Because the predicted etiological types of this study had four categories and the categories were imbalanced, we evaluated the performance of the model using micro-F1. micro-F1 is suitable for multi-classification problems and unbalanced data, and higher values represent better model performance. The calculation method for micro-F1 is as follows (taking four categories as an example):

- Total $Recall_{mi}=\frac{TP_{1}+TP_{2}+TP_{3}+TP_{4}}{TP_{1}+TP_{2}+TP_{3}+TP_{4}+FN_{1}+FN_{2}+FN_{3}+FN_{4}};$
- Total $Precision_{mi}=\frac{TP_{1}+TP_{2}+TP_{3}+TP_{4}}{TP_{1}+TP_{2}+TP_{3}+TP_{4}+FP_{1}+FP_{2}+FP_{3}+FP_{4}};$
- Calculate $micro\;F1\;score=2\;\frac{Recall_{mi}\times Precision_{mi}}{Recall_{mi}+Precision_{mi}}$

wherein TP_i_ refers to a true positive of class i; FP_i_ refers to a false positive of class i; TN_i_ refers to a true negative of class i; and FN_i_ refers to a false negative of class i.

## Results

### Brief Introduction of the Cases Selected for the Study

**Figure 1:**
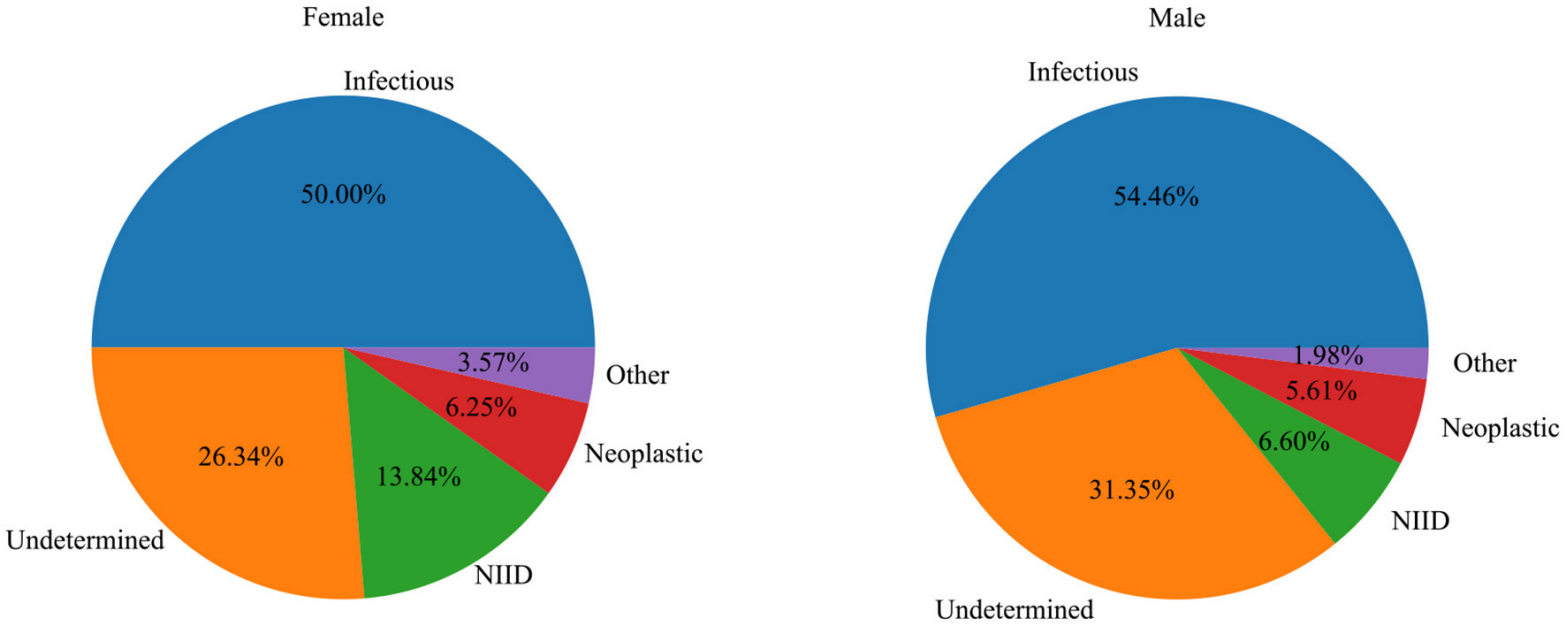
The relationship between the distribution of etiology and gender in patients with classic FUO.

A total of 527 patients with classic FUO were collected from seven medical institutions in Chongqing, including 303 men (57.5%) and 224 women (42.5%). Of the patients, 3.4% (*n* = 18), 19.2% (*n* = 101), 37.8% (*n* = 199), and 39.6% (*n* = 209) were <20, 20–39, 40–59, and ≥60 years, respectively. Table 1, Figure 1 show the distribution of classic FUO etiologies by age and gender, respectively.

**Table 1:** Percentages of causes of classic FUO ranked by age.

**Age**	**Infectious diseases (%)**	**Non-infectious inflammatory disease (%)**	**Neoplastic diseases (%)**	**Other diseases (%)**	**Undetermined (%)**	**Total**
<20	13 (72.2)	3 (16.7)	0 (0)	0 (0)	2 (11.1)	18
20–39	48 (47.5)	9 (8.9)	4 (3.9)	7 (6.9)	33 (32.8)	101
40–59	99 (49.8)	25 (12.6)	12 (6.0)	5 (2.5)	58 (29.1)	199
≥60	117 (56.0)	14 (6.7)	15 (7.2)	2 (1.0)	61 (29.1)	209
Total	277 (52.5)	51 (9.7)	31 (5.9)	14 (2.7)	154 (29.2)	527

**Figure 2:**
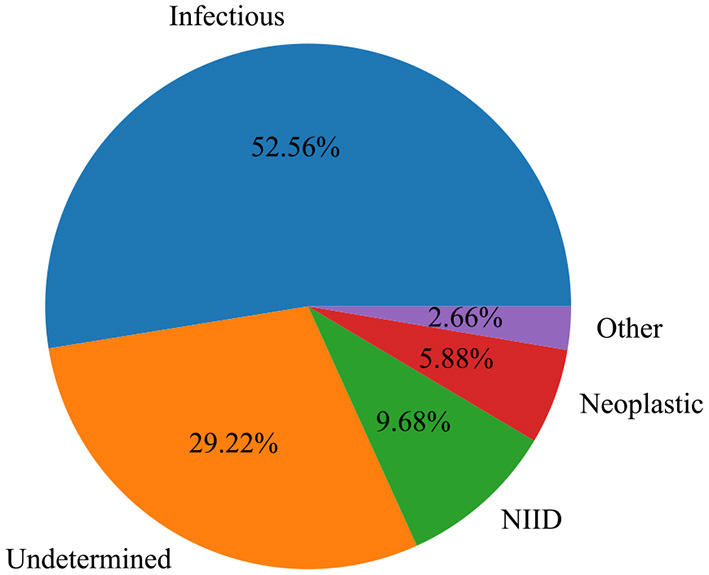
Etiology distribution of 527 patients with classic FUO.

Infectious disease (*n* = 277; 52.5%) and NIID (*n* = 51; 9.7%) were the most common causes of classic FUO (Figure 2). Infectious diseases included bacterial (*n* = 193), tuberculosis (*n* = 46), and other bacterial infections (*n* = 2) and viral (*n* = 21), fungal (*n* = 12), parasitic (*n* = 1), and other pathogen infections (*n* = 2). The most common NIIDs were hemophagocytic syndrome (*n* = 12), anti-neutrophil cytoplasmic antibody (ANCA)-associated vasculitis (*n* = 9), systemic lupus erythematosus (*n* = 9), and Adult-onset Still's disease (*n* = 7). Thirty-one cases (5.9%) were diagnosed as neoplastic diseases, of which eight cases were lymphoma. Other causes, such as subacute thyroiditis (*n* = 9) and drug fever (*n* = 3), were diagnosed in 14 patients (2.7%). A total of 29.2% (*n* = 154) of the patients remained undiagnosed at discharge (Table 2).

**Table 2:** Etiology distribution of 527 patients with classic FUO.

**Etiology**	***N* (%)**	**Etiology**	***N* (%)**
**Infectious diseases**	277 (52.5)	**Other pathogenic infections**	2 (0.4)
**Bacterial infections**	193 (36.6)	Mycoplasmal pneumonia	2 (0.4)
Respiratory system infection	116 (22.0)	**Non-infectious inflammatory disease**	51 (9.7)
Bloodstream infection	30 (5.7)	Hemophagocytic syndrome	12 (2.3)
Urinary tract infection	21 (4.0)	Systemic lupus erythematosus	9 (1.7)
Biliary tract infection	6 (1.1)	ANCA-associated vasculitis	9 (1.7)
Liver abscess	6 (1.1)	Adult onset still disease	7 (1.3)
Cellulitis	6 (1.1)	Sjogren syndrome	4 (0.8)
Pressure ulcers infection	2 (0.4)	Rheumatoid arthritis	2 (0.4)
Reproductive tract infection	2 (0.4)	Undifferentiated connective tissue disease	2 (0.4)
Infective endocarditis	2 (0.4)	Gouty arthritis	1 (0.2)
Umbilical infection	1 (0.2)	Dermatomyositis	1 (0.2)
Intra-abdominal infection	1 (0.2)	Takayasu arteritis	1 (0.2)
**Tuberculosis**	46 (8.7)	Crohn's disease	1 (0.2)
Pulmonary tuberculosis	35 (6.6)	Autoimmune hemolytic anemia	1 (0.2)
Extrapulmonary tuberculosis	11 (2.1)	macrophage activation syndrome	1 (0.2)
**Other bacterial infections**	2 (0.4)	**Neoplastic diseases**	31 (5.9)
Typhoid	1 (0.2)	Lymphoma	8 (1.5)
Brucellosis	1 (0.2)	Lung carcinoma	6 (1.1)
**Viral infections**	21 (4.0)	Hepatoma	5 (0.9)
HIV	10 (1.9)	Castleman's disease	3 (0.6)
Epstein-Barr virus	5 (0.9)	Acute myelogenous leukemia	2 (0.4)
Hepatitis B	4 (0.8)	Colon cancer	2 (0.4)
other viral infections	2 (0.4)	Myelodysplastic Syndrome	1 (0.2)
**Fungal infections**	12 (2.3)	Renal carcinoma	1 (0.2)
Candida albicans	2 (0.4)	Cholangiocarcinoma	1 (0.2)
Pneumocystis carinii pneumonia	2 (0.4)	Multiple myeloma	1 (0.2)
Crytococcus neoformans	2 (0.4)	Thyroid carcinoma	1 (0.2)
Pulmonary aspergillosis	1 (0.2)	**Other diseases**	14 (2.7)
Candida tropicalis	1 (0.2)	Subacute thyroiditis	9 (1.7)
Other fungal infections	4 (0.8)	Drug fever	3 (0.6)
**Parasitic infections**	1 (0.2)	Hyperthyroidism	1 (0.2)
Malaria	1 (0.2)	Necrotizing lymphadenitis	1 (0.2)
		**Undetermined**	154 (29.2)

### Test of the Difference in the Indexes of Patients With Classic FUO With Different Etiologies

There was a significant difference in the proportion of male and female patients with classic FUO among the four groups (χ^2^ = 8.24, *P* < 0.05). Male patients with FUO were common in the infectious and neoplastic disease groups, whereas female patients with FUO were common in the NIID and other diseases groups. There was a significant difference in age among the groups (H = 9.34, *P* < 0.05). The age of patients with tumor disease was the oldest [57.00 (43.50, 67.50)], whereas the age of patients with other diseases was the youngest [42.00 (32.50, 50.75)]. Regarding their past history, there was significant difference between patients with or without an history of blood transfusion and patients diagnosed with different types of causes (χ^2^ = 27.59, *P* < 0.001). There were significant differences in concomitant symptoms and physical examinations among the four groups (*P* < 0.05), except for nasal obstruction (χ^2^ = 2.66, *P* = 0.447), abdominal pain (χ^2^ = 5.79, *P* = 0.122), and splenomegaly (χ^2^ = 1.39, *P* = 0.708). In terms of laboratory tests, RBC (*F* = 6.97, *P* < 0.001), LDH (H = 12.37, *P* = 0.006), PCT (H = 15.69, *P* = 0.001), monocyte (H = 12.26, *P* = 0.007), lymphocyte (H = 8.51, *P* = 0.037), ALP (H = 9.83, *P* = 0.020), AST (H = 10.21, *P* = 0.017), and GGT (H = 8.70, *P* = 0.033) were performed. The results are shown in Tables 3, 4.

**Table 3:** Test of the difference of indexes (continuous indexes) in patients with classic FUO of different etiological types.

**Variable**	**Infectious diseases (%)**	**Non-infectious inflammatory disease (%)**	**Neoplastic diseases (%)**	**Other diseases (%)**	**χ^2^**	***P***
No. of cases	277	51	31	14		
**Gender**						
Male	165 (59.6%)	20 (39.2%)	17 (54.8%)	6 (42.9%)	8.24	0.041
Female	112 (40.4%)	31 (60.8%)	14 (45.2%)	8 (57.1%)		
**Operation history**						
Yes	109 (39.4%)	20 (39.2%)	13 (41.9%)	4 (28.6%)	0.76	0.858
No	168 (60.6%)	31 (60.8%)	18 (58.1%)	10 (71.4%)		
**History of blood transfusion**						
Yes	35 (12.6%)	8 (15.7%)	15 (48.4%)	1 (7.1%)	27.59	<0.001
No	242 (87.4%)	43 (84.3%)	16 (51.6%)	13 (92.9%)		
**Headache/consciousness disorders**						
Yes	66 (23.8%)	6 (11.8%)	3 (9.7%)	8 (57.1%)	16.32	<0.001
No	211 (76.2%)	45 (88.2%)	28 (90.3%)	6 (42.9)		
**Nasal obstruction**						
Yes	9 (3.2%)	3 (5.9%)	0 (0.0%)	0 (0.0%)	2.66	0.447
No	268 (96.8%)	48 (94.1)	31 (100.0%)	14 (100.0%)		
**Sore throat**						
Yes	25 (9.0%)	12 (23.5%)	1 (3.2%)	6 (42.9%)	23.96	<0.001
No	252 (91.0%)	39 (76.5%)	30 (96.8%)	8 (57.1%)		
**Abdominal pain**						
Yes	17 (6.1%)	6 (11.8%)	5 (16.1%)	2 (14.3%)	5.79	0.122
No	260 (93.9%)	45 (88.2%)	26 (83.9%)	12 (85.7%)		
**Arthralgia**						
Yes	19 (6.9%)	12 (23.5%)	3 (9.7%)	2 (14.3%)	14.09	0.003
No	258 (93.1%)	39 (76.5%)	28 (90.3%)	12 (85.7%)		
**Muscle pain**						
Yes	30 (10.8%)	12 (23.5%)	1 (3.2%)	0 (0.0%)	11.25	0.010
No	247 (89.2%)	39 (76.5%)	30 (96.8%)	14 (100.0%)		
**Rash**						
Yes	7 (2.5%)	5 (9.8%)	2 (6.5%)	2 (14.3%)	9.63	0.022
No	270 (97.5%)	46 (90.2%)	29 (93.5%)	12 (85.7%)		
**Lymphadenopathy**						
Yes	10 (3.6%)	9 (17.6%)	4 (12.9%)	0 (0.0%)	18.10	<0.001
No	267 (96.4%)	42 (82.4%)	27 (87.1%)	14 (100.0%)		
**Hepatomegaly**						
Yes	1 (0.4%)	3 (5.9%)	3 (9.7%)	0 (0.0%)	18.41	<0.001
No	276 (99.6%)	48 (94.1)	28 (90.3%)	14 (100.0%)		
**Splenomegaly**						
Yes	9 (3.2%)	2 (3.9%)	2 (6.5%)	0 (0.0%)	1.39	0.708
No	268 (96.8%)	49 (96.1%)	29 (93.5%)	14 (100.0%)		

**Table 4:** Test of difference of indexes (classification indexes) in patients with classic FUO of different etiological types.

**Variable**	**Infectious diseases (%)**	**Non-infectious inflammatory disease (%)**	**Neoplastic diseases (%)**	**Other diseases (%)**	**F/H**	***P***
Age [year, M (P_25_, P_75_)]	55.00 (42.00, 68.00)	51.00 (40.50, 60.50)	57.00 (43.50, 67.50)	42.00 (32.50, 50.75)	9.34	0.025
**Laboratory examination**						
Globulin (g/L, $\bar{x}\pm$s)	31.89 ± 6.95	33.20 ± 5.77	32.37 ± 7.66	33.47 ± 3.50	0.44	0.725
RBC (× 10^12^/L, $\bar{x}$ ± s)	3.82 ± 0.72	3.38 ± 0.68	3.38 ± 0.85	3.98 ± 0.67	6.97	<0.001
LDH [U/L, M (P_25_, P_75_)]	219.50 (157.75, 441.48)	340.00 (198.50, 629.50)	408.00 (244.45, 867.00)	191.00 (166.50, 251.58)	12.37	0.006
CRP [mg/L, M (P_25_, P_75_)]	61.90 (20.23, 115.11)	48.23 (10.21, 130.42)	112.78 (62.32, 152.64)	24.94 (8.38, 130.35)	6.59	0.086
PCT [ng/ml, M (P_25_, P_75_)]	0.21 (0.09, 0.77)	0.26 (0.10, 0.59)	0.43 (0.19, 1.89)	0.07 (0.05, 0.12)	15.69	0.001
ESR [mm/H, M (P_25_, P_75_)]	57.00 (29.50, 83.50)	69.00 (36.00, 94.50)	70.00 (38.75, 91.00)	49.00 (26.00, 78.75)	3.33	0.344
Monocyte [× 10^9^/L, M (P_25_, P_75_)]	0.47 (0.31, 0.66)	0.44 (0.15, 0.70)	0.71 (0.55, 0.99)	0.55 (0.41, 0.77)	12.26	0.007
Basophils [× 10^9^/L, M (P_25_, P_75_)]	0.01 (0.01, 0.02)	0.01 (0.00, 0.02)	0.01 (0.01, 0.02)	0.01 (0.00, 0.02)	0.53	0.913
Eosinophils [× 10^9^/L, M (P_25_, P_75_)]	0.04 (0.01,0.10)	0.02 (0.00,0.10)	0.03 (0.01,0.12)	0.02 (0.00,0.06)	1.67	0.645
Lymphocyte [× 10^9^/L, M (P_25_, P_75_)]	0.95 (0.61, 1.41)	0.79 (0.55, 1.15)	0.92 (0.61, 1.71)	1.43 (0.94, 1.61)	8.51	0.037
WBC [× 10^9^/L, M (P_25_, P_75_)]	7.20 (5.45, 10.51)	8.04 (5.20, 11.39)	10.27 (6.15, 16.94)	6.48 (5.52, 11.29)	3.15	0.370
ALP [U/L, M (P_25_, P_75_)]	83.30 (65.50, 119.25)	92.00 (71.00, 134.00)	110.60 (96.50, 208.00)	96.50 (78.75, 128.50)	9.83	0.020
PLT [× 10^9^/L, M (P_25_, P_75_)]	224.00 (172.00, 311.50)	214.00 (108.00, 303.00)	200.00 (86.00, 301.50)	334.00 (159.50, 408.00)	3.75	0.289
ALT [U/L, M (P_25_, P_75_)]	24.00 (13.00, 41.00)	29.00 (14.00, 53.60)	21.00 (14.00, 40.00)	26.00 (21.00, 36.00)	1.89	0.597
AST [U/L, M (P_25_, P_75_)]	26.00 (17.00, 44.00)	33.50 (23.00, 80.00)	30.00 (19.00, 53.00)	17.00 (14.40, 28.00)	10.21	0.017
GGT [U/L, M (P_25_, P_75_)]	46.00 (23.00, 108.00)	51.00 (27.00, 115.00)	77.00 (53.00, 196.00)	33.00 (28.00, 71.00)	8.70	0.033

### Prediction Model Performance

There were significant differences in the 18 characteristics including age and gender among the four different etiological types of patients with classic FUO. On the basis of the aforementioned indicators, five ML models were constructed and the whole dataset was included in the analyses. Table 5 shows the results of the five ML models. We mainly compared the sizes of the micro-F1 values. The micro-F1 value of each ML algorithm was the average of the five results in the 5-fold cross-validation. The micro-F1 of LightGBM was 75.8%, which was significantly higher than that of the other four ML algorithms (74.4, 73.4, 70.8, and 71.0%, respectively), and LightGBM has the best performance evaluation.

**Table 5:** Comparison of five ML models.

**Model**	**micro-F1 score, %**	**Recall_**mi**_, %**	**Precision_**mi**_, %**
RF	74.4	74.4	74.4
SVM	73.4	73.4	73.4
LightGBM	75.8	75.8	75.8
ANN	70.8	70.8	70.8
NB	71.0	71.0	71.0

**Figure 3:**
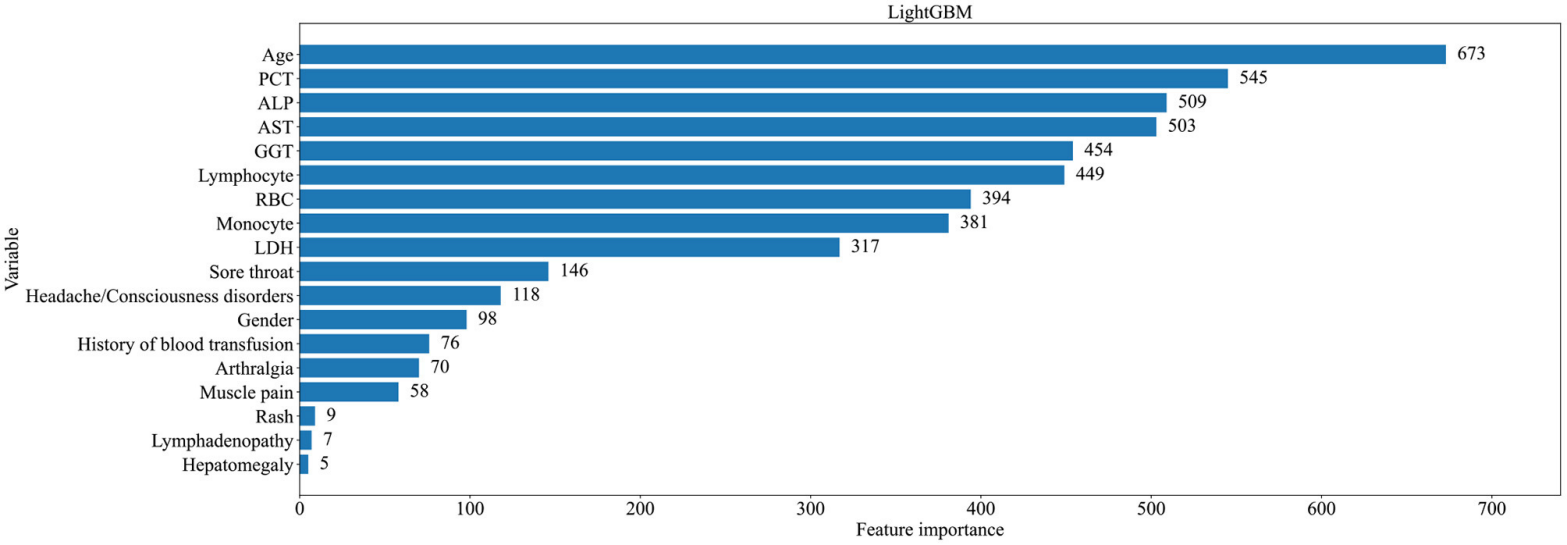
Ranking of importance of characteristics of patients with classic FUO in predicting all types of causes.

In order to better understand the contribution of each variable in our modeling results, we chose the LightGBM model with the best performance evaluation to present. Each variable was evaluated using Gini Importance, which is commonly used in ensembles of decision trees as a measure of a variable's impact in predicting a label that also takes into account the estimated error in randomly labeling an observation according to the known label distributions (23). Figure 3 shows the ranking of feature importance for all variables in the model. The results showed that age, PCT, ALP, AST, and GGT were the top five important features in the model, which made a great contribution to the prediction results.

**Figure 4:**
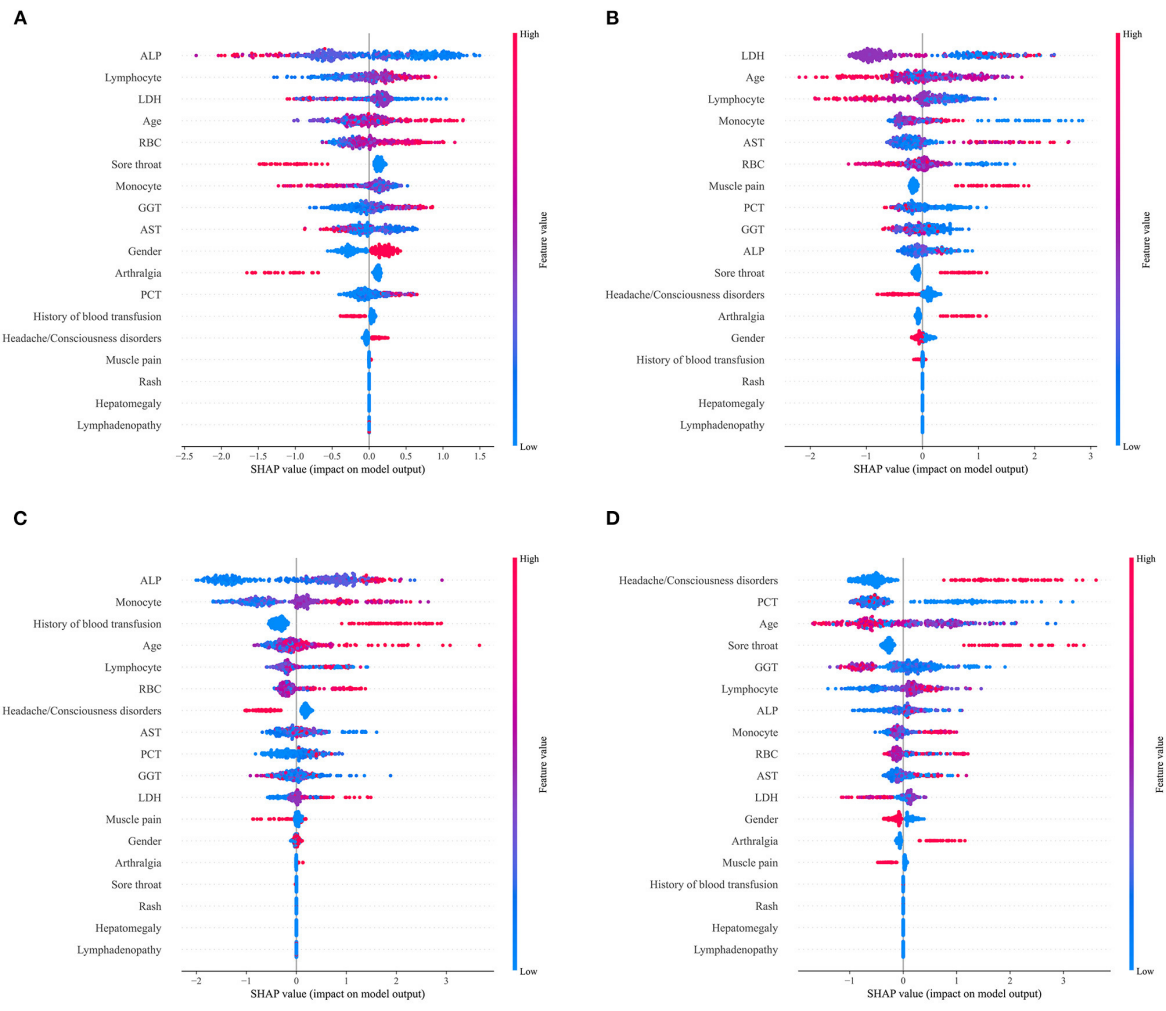
SHapley Additive exPlanations (SHAP) scores for identifying important features for the prediction of etiological types. **(A)** Infectious diseases; **(B)** NIID; **(C)** neoplastic diseases; and **(D)** other diseases. Colors indicate whether the value of the feature is high (red) or low (blue).

For the LightGBM model defined as the final prediction model, the relationship between each variable and the prediction outcome for the model is illustrated in Figure 3. To determine the most salient features that drove the model predictions, we calculated the SHapley Additive exPlanation (SHAP) values of the best-performing models for different etiological types. Figures 4A–D shows the important characteristics of each etiological type. For infectious diseases, age, lymphocyte, and RBC increased and ALP and LDH decreased in favor of the classifier to predict infectious diseases. For NIID, higher LDH, monocyte, and AST; younger age; and a lower lymphocyte were helpful to the classifier to predict NIID. For neoplastic diseases, higher ALP, monocyte, and lymphocyte; older age; and previous history of blood transfusion were conducive to the classifier to predict the cause of neoplastic diseases. For other diseases, accompanied by headache or disturbance of consciousness and sore throat symptoms, younger age and lower PCT and GGT were conducive to the classifier to predict the cause of tumor diseases. Other important features of each etiological type are shown in Figure 4.

## Discussion

The etiological distribution of 527 patients with classic FUO was analyzed retrospectively, and the patients were divided into five groups according to the final diagnosis, including the group with unknown etiology of fever at discharge. Analysis showed that infectious diseases were the most common cause of classic FUO, followed by NIID. These results are consistent with most of the previous research results at home and abroad (8, 24–26), and the reasons for this phenomenon may be related to the non-standard use of antibiotics and drug resistance leading to disease persistence and changes in the rule of fever type. There were also different findings between this study and previous studies. First of all, the proportion of tuberculosis infection in infectious diseases was 16.6% (46/277), which was significantly lower than that of Li's study (30%) (27) but similar to that of Zhai's study (17.6%) (24). This change may be related to the strengthening of public awareness of tuberculosis and the improvement of medical conditions in recent years. Conversely, with the improvements in diagnosis and treatments, most tuberculosis infections can be diagnosed clearly in the early stage, thus reducing the proportion of patients with tuberculosis with FUO. Second, this study showed that the proportion of HIV infection was 3.6%, which is higher than the previous research results (1%) (8), which may be related to the increase in floating populations, sexual attitudes and sexual behavior, sexual orientation changes, and other factors that increase the risk of HIV infection. This study found that the proportion of NIIDs was 9.7%, which was significantly lower than the results of Naito's research (30.6%) in 2013 (28). It may be due to the early use of relevant immunological indicators, which enabled the early diagnosis of autoimmune diseases with more typical symptoms, and no longer classified as classic FUO. In this study, neoplastic diseases accounted for 5.9% of classic FUO, which was significantly lower than 15%, as reported in the literature (1), which may be due to PET-CT and serum tumor markers that have been widely used in recent years (29, 30). Many malignant tumors can be diagnosed early, and the widespread use of early biopsy is also a reason for the reduction of neoplastic diseases with classic FUO. Among other diseases, subacute thyroiditis accounts for a considerable proportion (64.3%), which is in line with the findings of Popovska-Jovicić (31). Subacute thyroiditis rarely has persistent fever as the only clinical manifestation, generally have some related clinical manifestations (32), such as upper respiratory tract infection symptoms, weight loss, neck pain, fatigue, and anorexia. Routine thyroid color ultrasound, thyroid antibody tests, and thyroid function tests are rarely performed; therefore, thyroiditis is easily misdiagnosed as an upper respiratory tract infection. The manifestations of elderly patients with subacute thyroiditis are often not obvious, and other underlying diseases may also have clinical signs of subacute thyroiditis, or patients cannot provide a good medical history. A total of 154 patients (29.2%) whose reason for fever was still not clear at the time of discharge from the hospital and who had early discharge due to economic or other personal reasons, had shorter hospitalization times, which led to inadequate examination and diagnostic treatment during hospitalization. A tentative diagnosis followed, and eventually, they were classified in the unclear group, which may be the reason for the high proportion of patients in this group.

In this study, 373 patients with classic FUO were divided into four groups and the groups were compared. The study found that male patients were more common and older in the tumor group than in the other three groups, whereas female patients were more common and younger in the other disease groups than in the other three groups. Regarding past history, accompanying symptoms and physical examination of patients with classic FUO had some special clinical signs that could provide clinical clues worthy of attention. Among them, arthralgia was very common. In this study, patients with classic FUO in all four groups with different etiological types had symptoms of arthralgia. The most common rheumatic diseases were heterogeneous diseases with joint, bone, and muscle pain as the main symptoms, which could have involved internal organs (33). Infectious arthritis in infectious diseases and some hematological tumors also have manifestations of arthralgia (34, 35). Rash is an important concomitant sign in patients with classic FUO, which may provide an important clue for the etiological diagnosis of classic FUO. In classic FUO, most diseases can be accompanied by clinical signs of skin rash (4), including (1) infectious diseases, such as Epstein–Barr virus infection, typhoid fever, and infective endocarditis; (2) NIID, such as systemic lupus erythematosus, dermatomyositis, and adult-onset Still's disease; (3) neoplastic diseases, such as lymphoma; and (4) other diseases, such as drug fever. In this study, all patients in the infectious disease, NIID, and neoplastic disease groups had symptoms of lymphadenopathy. Lymph node enlargement is either localized or generalized (36). Localized lymphadenopathy involves a draining region, often caused by a non-specific inflammatory response of the tissue or organ in the draining region or by lymphatic metastasis of malignant tumors corresponding to the draining region. Direct invasion of infectious pathogens or immune response caused by infection, allergic or autoimmune diseases, and invasion of neoplastic diseases can lead to systemic lymphadenopathy. In laboratory examinations, we found that the levels of LDH, PCT, monocyte, ALP, and GGT in the neoplastic disease group were significantly higher than those in the other three groups, whereas the level of AST in the NIID group was higher than that in the other three groups, and the levels of RBC and lymphocyte in the other disease groups were higher than those in the other three groups. Among them, the higher PCT levels in the neoplastic disease group was an interesting finding. In general, increase concentration of blood PCT is associated with severe bacterial infection. However, the clinical interpretation of elevated PCT concentration in blood represents a great challenge in cancer patients since its values might be influenced by several factors such as the presence of metastasis or neuroendocrine function of malignant tissue (37). In these cases, PCT concentrations can be elevated regardless of infections, manifesting a poor specificity for bacterial infection. Matzaraki et al. (38) indicated that patients with solid tumors, metastasis, and no evidence of infection had markedly elevated PCT levels, especially those with generalized metastatic disease. Similarly, Liu et al. (39) show that in the absence of bacterial infection, PCT levels are elevated in patients with certain inflammatory conditions, such as Kawasaki disease, Adult-onset Still's disease and some cancers like medullary carcinoma of the thyroid and small-cell lung carcinoma.

On the basis of the aforementioned discussion, this study screened 18 indicators, such as gender and age, and constructed a clinical prediction model of the etiological types of patients with classic FUO. The indicators included in the model are all from the indicators reported in the consensus on current management of fever of unknown origin, which adds reliability to the model we constructed. We compared five ML algorithms, all of which were tested using the 5-fold cross-validation method. These five ML algorithms are widely used in clinical prediction model construction. For example, SVM learning is widely used in cancer genomics (40). Compared to other ML algorithms, SVM is very powerful in identifying subtle patterns in complex data sets. However, there are also some shortcomings, such as slow training speed and difficult to understand the internal operation. Ivanović et al. (41) constructed an ANN model to predict the lymph node status of clinical lymph node-negative breast cancer. ANN have the ability to adapt to variable interaction and non-linear correlation, but also have the constraints of opaque underlying model and difficult to explain (42). Yang et al. (43) constructed a response prediction model of breast cancer neoadjuvant chemotherapy based on NB algorithm. In their study, the NB algorithm showed higher predictive values than other algorithms. Each ML algorithm has its own advantages and disadvantages, but in our research data, the micro-F1 value of the LightGBM model was 75.8%, which was significantly higher than that of the other four ML algorithms. It is suggested that the LightGBM model has better predictive performance for the classification of etiological types of patients with classic FUO. LightGBM is a distributed gradient lifting framework based on a decision tree algorithm, which has high efficiency and performance in dealing with binary classifications and multi-classification problems (44–46). LightGBM is an ensemble algorithm developed by Microsoft, which is superior to other machine learning methods for disease diagnosis in many cases (45). Fundamentally, this is achieved by combining multiple base classifiers into an ensemble model by learning the inherent statistics of the combined classifiers and, hence, outperforming the single classifiers. In addition, the RF model also achieved a high accuracy, micro-F1 was 74.4%, which was second only to LightGBM in the results of this study. RF is recognized as one type of ensemble learning method and are effective for the most classification and regression tasks (47), which further illustrates the advantages of ensemble learning methods. In this study, 18 indexes related to the etiological diagnosis of classic FUO were ranked in descending order of importance. Among them, the ranking of laboratory indicators can provide doctors with decision support for laboratory examination to a certain extent. We also calculated the SHAP value of the best performance model according to the cause category for explaining the model, and we could clearly see the influence of the characteristics of each cause type on the output of the model. In the following research, how to deal with the imbalanced data set and the small sample size problem is worth considering, because these problems affect the performance of the prediction model to some extent. Data imbalance is widespread in the real world, especially in medical big data, which affects the accuracy of medical diagnosis classification learning algorithm to a certain extent. In order to solve the problem of poor performance of medical diagnosis learning algorithms due to the serious shortage of minority samples, Han et al. (48) proposed a distribution-sensitive oversampling method for unbalanced large data, including the distribution-sensitive minority sample selection algorithm and the minority sample synthetic algorithm of weight adaptive adjustment, which improves the quality of newly generated minority samples. This may be a way to improve the accuracy of the model. In addition, few-shot learning is also a research direction that we should pay attention to. Few-shot learning is such a research topic that studies how to learn a new concept from few training data of this concept and has received significant attention from the machine learning community (49).

Our study has several limitations. First, this was a retrospective study, which had its own shortcomings, such as information bias. Second, the prediction model may have lacked generality because the 30 variables are still too few and many other variables were omitted because of the loss of too many values. Therefore, we hope to include more patients and variables in future studies. In addition, 154 cases with unknown etiology of fever were not included in the model, which do exist in the real world. Therefore, the accuracy in reality may be lower, and these situations should be taken into account in future studies.

## Conclusions

In summary, this study retrospectively analyzed the clinical data of 527 patients with classic FUO from 7 medical institutions in Chongqing, discussed the differences of clinical indexes of 373 patients with classic FUO under 4 different etiological types, and introduced ML methods into the study of classic FUO to explore the application value of ML methods in the etiological diagnosis of classic FUO. The data of this study shows that infectious diseases are still the main etiological type of classic FUO. Based on 18 statistically significant clinical indicators such as gender and age, we constructed and compared 5 different ML algorithm models. The results show that compared with other algorithms, LightGBM is the best, and its micro-F1 value is 75.8%. We also use feature importance ranking and SHAP values to enhance the interpretability of the model. We believe that our model will provide clinicians with the most likely direction of etiological diagnosis in the diagnosis of classic FUO, assist clinicians to make reasonable decisions, improve the diagnostic accuracy of classic FUO, and reduce the misdiagnosis rate.

## Data Availability Statement

The original contributions presented in the study are included in the article/supplementary material, further inquiries can be directed to the corresponding author/s.

## Ethics Statement

The studies involving human participants were reviewed and approved by Medical Research Ethics Committee of Chongqing Medical University. Written informed consent for participation was not required for this study in accordance with the national legislation and the institutional requirements.

## Author Contributions

YY, CC, and KP participated in the research design and coordination and helped in drafting the manuscript. KP contributed in data acquisition. YY, CC, YL, ZZ, and LX analyzed the data. All authors contributed to the article and approved the submitted version.

## Funding

This study was supported by grants from the Natural Science Foundation of Chongqing (cstc2019jcyj-msxmX0027); the College of Medical Informatics, Chongqing Medical University, China, Student Research and Innovation Experiment Project (2019C011); the Philosophy and Social Sciences Innovation Team of Chongqing Medical University (ZX190101); and the Project of Innovative Research and Demonstration Base of Children's Medical Security in Children's Hospital Affiliated to Chongqing Medical University (NCRCCHD- 2019-HP-04).

## Conflict of Interest

The authors declare that the research was conducted in the absence of any commercial or financial relationships that could be construed as a potential conflict of interest.

## Publisher's Note

All claims expressed in this article are solely those of the authors and do not necessarily represent those of their affiliated organizations, or those of the publisher, the editors and the reviewers. Any product that may be evaluated in this article, or claim that may be made by its manufacturer, is not guaranteed or endorsed by the publisher.
